# A Survey of Resources and Nursing Workforce for Clinical Research Delivery in Paediatric Intensive Care Within the UK / Ireland

**DOI:** 10.3389/fped.2022.848378

**Published:** 2022-05-02

**Authors:** Julie C. Menzies, Claire Jennings, Rebecca Marshall

**Affiliations:** ^1^Paediatric Intensive Care Unit, Birmingham Women's and Children's NHS Foundation Trust, Birmingham, United Kingdom; ^2^College of Medical and Dental Sciences, Institute of Clinical Sciences, University of Birmingham, Birmingham, United Kingdom; ^3^Paediatric Critical Care Unit, Royal Manchester Children's Hospital, Manchester, United Kingdom

**Keywords:** research nurse, staffing, workforce, paediatric intensive care, study recruitment, research delivery, clinical academic

## Abstract

**Introduction:**

Clinical research within Paediatric Intensive Care (PICU) is necessary to reduce morbidity and mortality associated within this resource-intensive environment. With UK PICUs encouraged to be research-active there was a drive to understand how centres support research delivery.

**Aim:**

To identify the research workforce available within UK/Ireland PICUs to support clinical research delivery.

**Method:**

An electronic survey, endorsed by the Paediatric Critical Care Society (PCCS), was designed and reported in accordance with CHERRIES guidelines. The survey was distributed by email to all UK/Ireland Nurse Managers and Medical/ Nursing Research leads, aiming for one response per site during the period of April-June 2021. Only one response per site was included in analysis.

**Results:**

44 responses were received, representing 24/30 UK/Ireland sites (80% response rate). Responses from *n* = 21/30 units are included (three excluded for insufficient data). 90% (*n* = 19/21) units were research active, although only 52% (*n* = 11) had permanent research roles funded within their staffing establishment. The majority of units (*n* = 18, 86%) had less than two WTE research nurses. Resources were felt to be sufficient for current research delivery by 43% of units (*n* = 9), but this confidence diminished to 19% (*n* = 4) when considering their ability to support future research. The top barriers to research conduct were insufficiently funded/unfunded studies (52%; *n* = 11), clinical staff too busy to support research activity (52%; *n* = 11) and short-term/fixed-term contracts for research staff (38%; *n* = 8).

**Conclusion:**

Despite the perceived importance of research and 90% of responding UK/Ireland PICUs being research active, the majority have limited resources to support research delivery. This has implications for their ability to participate in future multi-centre trials and opportunities to support the development of future medical/nursing clinical academics. Further work is required to identify optimum models of clinical research delivery.

## Introduction

There is a growing body of evidence which illustrates that research active hospitals not only have better patient outcomes (1) and increased levels of patient satisfaction (2), but also improved staff retention, staff satisfaction and improved organisational efficiency (3). In a study of 129 English NHS Hospital Trusts (4) an increase in research activity was correlated with a better Care Quality Commission (CQC) rating and lower mortality rate obtained from Summary Hospital-level Mortality Indicator (SHMI) scores. In addition, the variable most associated with improved CQC rating and SHMI mortality score was the number of patients recruited into interventional studies. With recognition that research is a vital component of a high quality service, the Care Quality Commission (CQC) well-led framework now incorporates questions to ensure that clinical research is embedded as a founding principle in all specialties (5).

Conducting clinical research involving critically ill children is vital because PICU is a high-cost, resource-intensive environment with a sparse evidence base (6). Theoretically it is an ideal environment for the conduct of clinical research with, greater physiological monitoring data and detailed clinical records with a huge potential for lifelong clinical and cost-effective analysis (7, 8). However figures indicate that recruitment of critically ill children and young people to research is low. Less than 1% of paediatric patients who undergo surgery for a congenital cardiac disease are recruited to cardiac surgical trials (9) and fewer than 1 in 100 admissions to PICU globally are recruited to a clinical trial, compared to one in 10 adults in ICU (10). High recruitment rates to national or international paediatric oncology trials (over 70%) has been attributed to increased survival and quality of life for children diagnosed with cancer (11). Clearly there is a significant amount of work required to offer the same opportunities to children and young people who experience critical illness or injury.

Failure to recruit to target is a challenge for clinical trials in all clinical specialties. A review of 122 trials (1994-2002) found that less than a third (31%) of the trials achieved their original patient recruitment target (12). This increased to 55% in 2013 (trials funded 2002-2008) (13) and 56% in 2016 (2004–2016) (8), however trials in emergency care and ICUs are four times more likely to be prematurely discontinued due to slow patient recruitment (14). This research waste is not only unethical, but also fails to advance knowledge and practise to support care for critically ill children and young people. If research is to be the standard of care for critically ill children and their families (15) then understanding current service provision is vital. The Paediatric Critical Care Society (PCCS) (16) have developed UK standards (17) and these highlight the importance of research in service provision. The society offers a research study group (PCCS-SG) with membership comprised of research-active clinicians and research delivery staff to help oversee all national multi-centre trials and facilitate collaboration (18). With PCCS-SG keen to facilitate more UK PICUs centres to be research-active, there was a drive to understand more about how centres support critical care research delivery.

Much of the day to day management and conduct of research is undertaken by Clinical Research Nurses; nurses specifically employed to recruit and care for patients taking part in clinical research (19). Their role has been identified as pivotal for successful research delivery and safety monitoring (19–24). Despite recognition of the importance of Research Nurses, 25% of Trusts surveyed were unsure how many were working within their organisation (25) and the national numbers in 2021 is currently unknown (26). In addition, there is a lack of consensus on optimum study to staff ratios to aid workforce planning and wide variation in research delivery team staff structures (25). All of this creates challenges to articulating the contribution of research nurses to the delivery of quality clinical research, which needs to be addressed (27). We were also interested in capturing information about units which offered opportunities for nurses to progress as nurse researchers; nurses who develop their own research ideas and conduct research independently (28) and clinical academics; healthcare professionals who combine clinical and research responsibilities within their role (29).

I. current research nurse staffing on research-active PICUs
II. attitudes towards research delivery and research nurses working within PICU
III. the barriers to research delivery across PICU
IV. the opportunities to support nurse-led research within PICU

## Materials and Methods

The research design was a survey of all UK and Republic of Ireland (IRL) PICUs. This method was selected to gather general quantifiable information at one point in time on research workforce and staffing (30). No formal ethical approval was required as the work was categorised as staff research (31).

No validated survey relevant to the project aims and objectives was available; therefore an electronic questionnaire to conduct the survey was developed by the research team (see Supplementary Material). Questionnaire content was generated from a number of sources: (i)aspects research delivery teams consider when reviewing capacity for new studies, (ii) questions which have been raised about research capacity and capability within PCCS-SG (18), (iii) questions raised within clinical academic career forums locally and nationally. Face and content validity of the questionnaire was achieved by pilot testing the questionnaire with a similar sample population at two sites (three research nurses, two nurse managers and two research leads) to ensure the questions were clear and understandable. Minor changes were made to the survey introduction, to the wording of six questions to enhance clarity and additions made to drop-down options to ease completion. Translation in to other languages was not considered necessary as all respondents were health care professionals practising within the NHS. The work was also subject to expert panel review by PCCS-SG for assessment of readability, content and feasibility, further enhancing the face validity (30) and was subsequently endorsed for dissemination.

The 30 NHS sites were identified from the Paediatric Intensive Care Audit Network (PICANet) report (32). The sample population was defined as being PICU Nurse Managers or Matrons (summarised as Nurse Managers from here on) or PICU Research leads (Medical or Nursing). A link to the electronic survey was initially distributed via email to members of the PCCS Managers group by the group Chair in April 2021, with a reminder in May 2021. A second strategy was then initiated with an invitation email distributed by PCCS-SG Chair to PICU Medical and Nursing Research Leads (May 2021). A third strategy was to invite members of PCCS-SG Research Nurse Forum via an email from the forum chairs (JM/CJ) to reach research nurses working within PICU and encourage participation from sites where there had been no response. The final strategy was to purposively contact non-responding sites to identify the Nurse Manager or research leads and prompt completion (June-July 2021). The covering information in the email identified that informed consent was indicated by participant completion of the questionnaire. Invitation to participate came directly through relevant group Chairs (Managers group and PCCS SG). The researchers did not have access to personal data or contact details about potential participants. Demographic data about the participants was limited to the unit they were responding on behalf of and their role. No personal data was collected about participants. To minimise incomplete responses, options were included for “not known” or “not relevant”, where appropriate. The survey was estimated to take 10–12 min to complete and included 24 questions which were dichotomous questions about resources, likert scales to capture views on importance or multiple-choice questions with boxes for any additional information (all compulsory to complete) and two open-ended, free text questions. Respondents were able to review and amend responses if desired; although there was no option to save and return to the survey at a later date. All surveys with data beyond just demographic data were eligible to be included. Where multiple responses per unit were received, responses were compared and contrasted to check for consistency, but only one response per site was included. Responses were prioritised in order: Nurse Managers, Nursing Research leads, research-active/Clinical Academic Nurses, Medical Research leads/research-active Doctors, research nurses. No incentives were offered to respondents. The results of the questionnaires were anonymised, with allocation of a unique site number, stored on a secure NHS drive and analysed and reported using descriptive statistics. Where free-text was provided examples are reported in the results to add clarity or additional insight. The study was designed in accordance with the CHERRIES guidelines (33). Although the survey was not administered on the internet, many of the CHERRIES items are valid for surveys administered via email (33). We have also referred to the SURVEY guidance for reporting survey studies robustly (30).

## Results

### Demographics

In total 44 survey responses were received, representing 24/30 UK/Ireland sites (80% response rate), with responses from all countries in the UK and Ireland. Ten sites contributed more than one response and three responses were excluded for insufficient data, therefore responses from 21/30 units (70%) are included within the analysis; all fully completed. This response rate was viewed as sufficient to draw conclusions (34). See Supplementary Material for site responses. Included responses were from our target participant groups; eight (38%) PICU Nurse Managers, five (24%) Nursing Research Leads, one (5%) Medical Research Lead, three (14%) “research-active” nurses [Nurse Principal Investigator (s)/Nurse Researcher (s)/Lecturer Practitioner (s)] (see Table 1). 90% (*n* = 19/21) of PICUs were research active and research activity reflected all the PCCS-SG documented multi-centre studies currently in progress, national COVID-research, as well as a number of smaller single centre studies. Unit research activity was a mean of 4.9 studies.

**Table 1:** UK/ IRL units survey responses.

**Respondent**	**All responses**	**Included responses**	**Unit research active**	**Research is very - extremely important**
Nurse Manager / Matron	17^a^, ^b^	8 (38)	7/8	8/8
Research Lead (Nurse)	5	5 (24)	5/5	4/5
Research active Nurse	3	3 (14)	2/3	2/3
Research Nurse	7	2 (10)	2/2	2/2
Research Active Dr	4	2 (10)	2/2	1/2
Research Lead (Dr)	4	1 (4)	1/1	1/1
Nurse	4	0 (0)	0	0
**Total**	**44**	**21 (100)**	**19/21 (90%)**	**18/21 (86%)**

a *3 were incomplete,*

a *>1 Matron/ nurse managers completed*.

### Research Delivery

Research was identified as important in service provision with 18/21 units (86%) stating that being able to offer patients and families the opportunity to participate was very – extremely important to them (Table 1). The majority of respondents (*n* = 20/21, 95%) had access to staff to support research delivery in their unit, with the main staff group responsible for research delivery being research nurses (*n* = 14, 70% of units) (see Table 2). Within those units with access to research nurse(s), four PICUs (29%) had less than one Whole Time Equivalent (WTE) (37.5 h/week) research nurse time, seven units (50%) had one-two WTE and only three units (21%) had above two WTE research nurses. The banding of research nurses within these 14 units ranged from Bands 5–8, although the majority of units employed, or had access to, Band 6 Research Nurses (*n* = 12, 86%). Career progression beyond this appeared to be limited, with only five units employing or having access to a Band 7 Research Senior Nurse and only one unit employed a Band 8a Nurse. 52% of units (*n* = 11) offered research nurse secondments or rotational posts where clinical nurses were offered the opportunity to rotate to a research post and gain research skills.

**Table 2:** Research delivery staffing across UK/ IRL PICUs.

	**Number of units (%)**
**Research workforce**	
Research nurse (s)	14 (70)
Research coordinator (s) / facilitator (s)	5 (25)
Research fellow	5 (25)
Admin assistant	3 (15)
Audit nurse	2 (10)
Research practitioner	1 (5)
Post doc research fellow	1 (5)
Reader	1 (5)
Phd student	1 (5)
**Research nurses whole time equivalent (wte)**	
0	7 (33)
<1	4 (19)
1–2	7 (33)
2–4	2 (10)
4+	1 (5)
**Total**	**21 (100)**
**Banding of research nurse posts**	
Not applicable	7 (33)
5	3 (21)
6	12 (86)
7	5 (36)
8	1 (7)
**Total**	**21 (100)**
**Secondment / rotational posts available**	
Yes	11 (52)
No	10 (48)
**Total**	**21 (100)**
**Research nurse provision**	
Picu embedded service	11 (52)
Clinical research nurses from wider trust (with picu experience)	6 (29)
Clinical research nurses (with no picu experience)	3 (14)
Clinical research nurses (clinical research network)	2 (10)
Clinical research practitioners / facilitators	2 (10)
Don't know^a^	1 (5)
**Line management**	
Trust research team (external to picu)^b^	8 (38)
Nurse manager (picu)^b^	5 (24)
Research nurse band 7 (within picu)	3 (14)
Clinical research facility (external to picu)	2 (10)
Not applicable	3 (14)
**Total**	**21 (100)**
**Research cover**	
Weekends: standard	2 (10)
Weekends- short term / *ad-hoc* basis	4 (19)
Outside core office hours: standard	0
Outside core office hours (8–5pm): short term / *ad-hoc* basis	8 (38)
On-call: standard	0
On-call: short term /*ad-hoc* basis	7 (33)
**Importance of research nurses**	
Very important	18 (86)
Somewhat–fairly important	3 (14)

a *This unit reported having no research open at the time of the survey,*

b *Co-line management between PICU Nurse Manager and Trust Research team*.

Research delivery staff were not always funded as part of “core” PICU business. Only 62% (*n* = 13) of units reported having staff funded within the workforce establishment to deliver research studies and only 11 units (52%) had **permanent** research roles funded within their staffing establishment. An embedded research team was seen by some units as key to the unit being able to support research activity:

“*We were not consistently research active until 2017 when we established our in-house research team.”* (Centre 16)

However several participants commented that finding funding for an embedded research service was challenging;

“*Lack of funding for a PCCU research nurse post”* (Centre 15).

“*There are NO PICU funded Research posts at the moment.” (Centre 14)*

Units which did not have access to PICU based research nurses (*n* = 10) accessed a wide range of research nurses to support research delivery. Six units accessed research nurses from the wider Trust with PICU experience, three accessed research nurses with no PICU experience and two had access to research nurses employed by the Clinical Research Network (PICU skill set not specifically stated). Three units reported using multiple resources to support activity. Units were asked whether they felt resources were sufficient for current research delivery. 43% of units (*n* = 9) felt they were, but this confidence diminished to 19% (*n* = 4) when considering their ability to support future research.

### Research Cover

Outside of “traditional” office hours (8–5pm) only two units offered weekend cover as standard practise and a further four could provide this on an *ad-hoc* planned basis. No units offered research cover outside of core office hours or an on-call service as standard practise. There were reflections that this was only possible because of the flexibility of the research workforce, particularly an embedded PICU research service:

“*We manage a lot of studies with minimal research nurse staffing. The research team have gone above and beyond to support studies during evening and weekends.” (Centre 16)*

If a study required randomisation out of office hours, this responsibility was most likely to be covered by Registrars/Advanced Nurse Practitioners (*n* = 14), Consultants/PI (*n* = 13) and/or clinical nurses (*n* = 12).

### Barriers and Facilitators to PICU Research Activity

Four sites reported having no barriers to research (see Table 3). The top three barriers to research conduct amongst the other 17 sites were unfunded studies (*n* = 8) and insufficient funding (*n* = 6) which were combined, *n* = 11 sites (52%), clinical staff too busy to support research activity (52%; *n* = 11) and short-term/fixed-term contracts for research staff (38%; *n* = 8).

**Table 3:** Barriers to research delivery.

	**Number of units (%)**
**Perceived barriers to research conduct**	
Clinical staff too busy to support research	11 (52)
Study unfunded	8 (38)
Insufficient funding	6 (29)
	**Combined** **=** **11 units (52)**
Short-term/ fixed contracts	8 (38)
Lack of invitation to participate	5 (24)
Lack of Principal Investigator	5 (24)
Recruiting staff to research posts	4 (19)
No barriers	4 (24)
Other	3 (14)
Not relevant research to our unit	2 (10)
Unit culture	2 (10)
Not important studies for our unit	1 (5)
**Sufficient resources for current research delivery**	
Yes	9 (43)
No	10 (48)
Unsure	2 (9)
**Total**	**21 (100)**
**Sufficient resources for future? research delivery**	
Yes	4 (19)
No	14 (67)
Unsure	3 (14)
**Total**	**21 (100)**

Several respondents commented on the positive attitude of clinical staff towards research but highlighted that there was limited capacity to support research:

“*Clinical staff do want to support research but often staffing clinically is insufficient to complete all the research procedures.”* (Centre 18)

The challenge of short-term contracts for research staff was highlighted by several respondents. This has issues for developing and retaining research nurse expertise:

“*Challenges recruiting experienced research staff. Once you've trained up inexperienced staff their contract ends.”* (Centre 6)

When respondents were asked what they felt their unit did well or they felt proud of in relation to research there were 18 responses. These reflected pride in both the research team (where they were available) and pride in the clinical team. Positive comments about the research team reflected their drive and passion, teamwork with clinical colleagues and despite low staffing their achievements with recruitment, funding and implementation of research. Pride in the clinical team was reflected in comments about how they embraced and supported research to help overcome the challenge of limited resources and worked collaboratively with research colleagues:

“*Despite a lack of staff we're currently undertaking non-NIHR studies - supported by clinical staff.”* (Centre 19)

### Research Careers

Respondents were asked about line management for research staff. This free-text answer generated a large number of responses, reflecting the multiplicity of roles and approaches to line management. In five units (24%) this was provided by the clinical PICU Nurse Manager. At 13 sites line management was undertaken by a senior research nurse – three (14%) offered this internally through PICU Nursing Research lead and 10 units (48%) were line managed by a team external to PICU, most commonly a Trust clinical research team. The role of a research nurse was felt to be very important by 86% (*n* = 18) of respondents. Respondents were also supportive of research designed and led by nurses, with 67% (*n* = 14) ranking this as very important (see Table 4). The main way units supported staff to develop was to support dissemination of conducted work, with 62% (*n* = 13) supporting staff to present work locally and 71% (*n* = 15) of units supporting staff to present at conferences. Units also supported staff to apply for personal research awards (*n* = 9, 43%) or research internships (*n* = 7, 33%). However there were challenges to this provision, mostly related to time. One participant commented that although there was support in principle, funded time was not available:

**Table 4:** Clinical academic development.

	**Number of units (%)**
**Importance of nurse-led research**	
Very important	14 (67)
Somewhat–fairly important	7 (33)
**Total**	**21 (100)**
**Available opportunities**	
Support to present at conferences	15
Support to present work locally	13
Support / time to write a paper	9
Support to apply for a personal research award e.g. MSc/PhD	9
Support to apply for a research internship	7
Funded time to undertake a piece of work/project	6
Undertake a research placement outside of PIC/PCC	5
Funded time as a 'research champion'	3
Other	0
**Career opportunities**	
No, but I would like there to be	8
Yes, clear pathways including on PIC/ PCC	5
No and I am aware of Nurses who have left because of this	4
Yes, clear pathways, but outside of PIC/PCC	4
**Total**	**21 (100)**

“*Any projects/ presentations nursing staff wish to do are in their own time.”* (Centre 4)

Only 24% of units reported there were clear pathways for nurses interested in research *within* PICU (*n* = 5, 24%). Six respondents reported there were clear pathways, but *outside* of PICU. Two respondents reported that that there were no opportunities within their unit, and were aware of nurses who had left because of this and eight units reported there were no opportunities but would like there to be:

“*Research nurse pathway clear, but not nurse researcher pathway”* (Centre 16)

## Discussion

### Research Delivery in PICU

With an average of 55 admissions per day to UK/IRL PICUs, 20,000 admissions per year and reported rates of bed occupancy over 80% across the UK/IRL (32) the scope for research activity within PICU provision is large. Although 90% of UK /IRL PICUs report being research-active, only 52% of these units have research staff on permanent contracts and over three-quarters of PICUs are delivering research on two or less WTE research nurses. Achieving research recruitment similar to specialities such as paediatric oncology (11) therefore seems challenging on current resources. With sparse research staffing there could also be compromise to quality and safety monitoring [16; 17; 18; 19], particularly as clinical nurses have limited capacity to support research-related activities/ procedures in a timely manner (35). Other studies have also identified that research staff can feel concerned about adding workload to departments already at capacity (36) which could limit research activity. With only 19% of units perceiving that they had sufficient resources to support future research, further investment in research capacity is required to facilitate increased research activity.

With the number of patients recruited into interventional studies identified as the variable most associated with improved CQC rating and reduced mortality rates (4), research is recognised as a vital component of a high quality service (3). The results of this survey suggest that this is currently not prioritised sufficiently within PICU. As hospital care is viewed as a 24 h a day service; there are questions about research provision often being limited to office hours and week days. No unit within this survey provided out-of-hours cover or an on-call service and only two units provided weekend research cover as part of standard service provision. If randomisation was required outside of office hours this responsibility would fall on clinical teams. Currently there is little published evidence to support staffing ratios or models of working and there is a lack of evidence concerning how best to structure clinical research teams within acute trusts (37). Workforce planning for nurse staffing within PICU states there is a requirement of at least 7.01 nurses per bed (38). An equivalent standard for research is not currently available. PCCS Standards 2021 (17), state that PICUs should actively participate in research relating to paediatric critical care; however this may not be applicable if appropriate support for research is not available locally. If research delivery is to be prioritised as core business, recommendations could extend to setting standards for this research support.

### Staffing and Contracts

The challenges of short term research contracts and insufficient funding identified by study participants as two of the biggest challenges faced within the PICU setting, are similar to concerns identified in a national study of research delivery practise (25). Fixed term contracts were commonly reported and whilst there were some reports of this being an opportunity to “try out” a new role and develop experience, more often they were negatively perceived in terms of financial stability and job security. The current NHS climate means research delivery can be difficult and often overlooked as it is not perceived as a priority (37). Many services fund research nurses through reactive recruitment; advertising and appointing to posts only when study funding was confirmed. This can impact negatively impacted on team morale, affecting sites ability to open studies in a timely manner, recruit to national targets and delays to being able to offer patients the opportunity to participate in research (25).

For the 10 sites that did not have any permanent research nurse posts, five were covered by research nurses from within the wider Trust or Clinical Research Network with no specific PICU experience. Whilst a multi-speciality team of research nurses who can provide cross-coverage for any study is considered useful and is often the model employed within organisations, this can be challenging for successful study management. A lack of clinical knowledge and competence, lack of confidence covering the PICU speciality, lack of confidence in the staff member by the clinical team and the risk of patient safety issues have all been identified as risks associated with “generic” research nurse cover (25, 35). McNiven et al. (39) described how research nurses were “boundary spanners”; offering the benefits of health care professional knowledge and familiarity with clinical working, as well as research ability. Several studies have highlighted that research activity is enhanced when research nurses are better integrated or embedded into the area (37) as successful clinical research is dependent on good working relationships between research and clinical teams (35). However this is challenging for many services to fund and sustain.

### Development of Clinical Academic Nurses

The majority of respondents (*n* = 19, 90%) identified that they felt it was fairly to very important to develop more nurse-led research and 76% (*n* = 16) provided opportunities to support nurse development. This support most commonly took the form of providing opportunities to present work. Whilst this is an important first step, having protected, funded time to develop and / or conduct research is one of the most important resources to developing clinical academic nurses (40–42). However, only six units (26%) said “funded time” was an opportunity they could offer and further detail was not provided as to the level and extent of this support. This situation is consistent with the national picture in the UK. Despite a national drive to increase the number of Nurses, Midwives and Allied Health Professionals in clinical academic positions by 2030 (43), this situation has been slow to change and there is a lack of sustained and cohesive implementation of clinical academic research pathways (44, 45).

There is recognition that research engagement by clinical nurses is an important precursor to this goal. Exposing nurses to research may help them to develop a more curious approach to their own practise, increasing their motivation towards research engagement (46). In addition the role of clinical research nurse is considered part of a career path that with advanced education and experience could extend to the role of nurse scientist or independent nurse researchers (39, 47). Creation of these opportunities and support for career development are dependent on strong senior nurse leadership (37, 40). Line managers play a large part in supporting career aspirations via appraisal and promotion mechanisms and through supporting opportunities for involvement. However, there was wide variation reported in how line management was provided. Many senior clinical managers have had little exposure to research, can feel unsure about how best to support the development of others or can lack understanding of the importance of research (37, 48). Line management within an external research team offers understanding about research, but may not facilitate staff wanting to progress within PICU into future clinical academic posts. In the UK PICU community, there are a growing number of clinical academic nurses (49), but with only 24% of PICUs reporting they offer career opportunities for nurses interested in research, further work is required to talent spot and support others.

## Limitations

The main limitations of the survey reflect those commonly identified about self-report questionnaires; that the 24-item tool offered a relatively superficial insight, there was no opportunity to seek clarification about responses and the survey may have been completed by participants who did not have the best insight into the phenomenon of interest (30, 33, 50). The predominant nature of closed-questions within the survey could have restricted participants' feedback. This approach was adopted to avoid burdening respondents (30) and promote a good response and completion rate. As the facility to provide free text was utilised and an overall response rate of 80% was achieved we feel this approach was appropriate and participants were able to respond and offer all appropriate information. Despite significant efforts, only 38% of responses were from PICU Nurse Managers. However as a further 43% of responses were from Nursing/ Medical research leads or Clinical Academic / Principal Investigators we feel confident that people with up to date knowledge about research staffing and delivery completed the survey. A further limitation was that the survey platform did not offer the option to “save and return”, necessitating completion in one response. This could have contributed to the 10 incomplete responses. However, with an overall response rate from 80% of sites, we feel this did not significantly affect completion.

## Conclusion

Despite the perceived importance of research and 90% of responding UK/IRL PICUs being research active, the majority have limited resources to support research delivery. This has implications for their ability to contribute to an increased research portfolio and increased research delivery, as well as opportunities to support the development of future medical and nursing clinical academics. Research in critically ill patients is challenging; research in critically ill children even more so (7), therefore we need to ensure that we have access to a skilled, knowledgeable workforce who feel confident working within the PICU context. PCCS standards identify that 7.01WTE nursing staff are required per PICU bed (38); however no standards are currently set for research delivery. If research is to become the standard of care for every patient admitted to PICU (15) then significant investment is required to increase the research delivery workforce.

## Data Availability Statement

The original contributions presented in the study are included in the article/Supplementary Material, further inquiries can be directed to the corresponding author/s.

## Ethics Statement

Ethical review and approval was not required for the study on human participants in accordance with the local legislation and institutional requirements. Written informed consent for participation was not required for this study in accordance with the national legislation and the institutional requirements.

## Author Contributions

JM developed the study concept and completed the initial and subsequent draughts of the manuscript, with manuscript edits from CJ and RM. JM, CJ, and RM developed and piloted the survey, facilitated distribution, and contributed to data interpretation. All authors reviewed and approved the final manuscript.

## Author Disclaimer

The views expressed in this article are those of the author and not necessarily those of the NIHR, or the Department of Health and Social Care.

## Conflict of Interest

JM is a National Institute for Health Research (NIHR) Senior Nurse and Midwife Research Leader. The remaining authors declare that the research was conducted in the absence of any commercial or financial relationships that could be construed as a potential conflict of interest.

## Publisher's Note

All claims expressed in this article are solely those of the authors and do not necessarily represent those of their affiliated organizations, or those of the publisher, the editors and the reviewers. Any product that may be evaluated in this article, or claim that may be made by its manufacturer, is not guaranteed or endorsed by the publisher.
